# Increases in Pediatric Antiretroviral Treatment, South Africa 2005–2010

**DOI:** 10.1371/journal.pone.0044914

**Published:** 2012-09-13

**Authors:** Sandeep D. Patel, Elysia Larson, Thobile Mbengashe, Heidi O’Bra, J. W. Brown, Thurma M. Golman, Jeffrey D. Klausner

**Affiliations:** 1 U. S. Indian Health Services, Belcourt, North Dakota, United States of America; 2 American Schools of Public Health at U.S. Centers for Disease Control and Prevention, Pretoria, South Africa; 3 National Department of Health, Pretoria, South Africa; 4 Division of Global HIV/AIDS, U.S. Centers for Disease Control and Prevention, Pretoria, South Africa; 5 U.S. Agency for International Development, Pretoria, South Africa; University of Massachusetts Medical School, United States of America

## Abstract

**Background:**

In South Africa in 2010, about 340,000 children under the age of 15 were infected with HIV. We describe the increase in the treatment of South African pediatric HIV-infected patients assisted by the President’s Emergency Plan for AIDS Relief (PEPFAR) from 2004 to 2010.

**Methods:**

We reviewed routine program data from PEPFAR-funded implementing partners among persons receiving antiretroviral treatment age 15 years old and less. Data quality was assessed during the reporting period by program officials through routine analysis of trends and logic checks. Based on UNAIDS estimated mortality rates of untreated HIV-infected children, we calculated the number of deaths averted and life-years gained in children under five receiving PEPFAR-assisted antiretroviral treatment.

**Results:**

From October 2004 through September 2010, the number of children newly initiated on antiretroviral treatment in PEPFAR-assisted programs increased from 154 to 2,641 per month resulting in an increase from 2,412 children on antiretroviral treatment in September 2005 to 79,416 children in September 2010. Of those children who initiated antiretroviral treatment before September 2009, 0–4 year olds were 1.4 (95% CI: 1.3–1.5) times as likely to transfer out of the program or die as 5–14 year olds; males were 1.3 (95% CI: 1.0–1.7) times as likely to stop treatment as females. Approximately 27,548 years of life were added to children under-five years old from PEPFAR-assisted antiretroviral treatment.

**Conclusions:**

Pediatric antiretroviral treatment in South Africa has increased substantially. However, additional case-finding and a further acceleration in the implementation of pediatric care and treatment services is required to meet the current treatment need.

## Introduction

South Africa has the world’s greatest burden of Human Immunodeficiency Virus (HIV) infection with an estimated 5.7 million persons infected with HIV in 2010, including 340,000 children under the age of 15 years [1], [2]. South Africa’s under five mortality rate increased substantially from 1996–2001 [3]. Most of those deaths are caused by conditions that are either preventable or treatable, such as Acquired Immune Deficiency Syndrome (AIDS) (35%), neonatal causes (30%) and pneumonia and diarrhea (17%) [4]. The South African Government (SAG) launched a national strategy to address the HIV epidemic in children and adults in 2003 [5]. The SAG Guidelines for the Management of HIV in Children at that time (in 2004) outlined criteria for commencing antiretroviral therapy (ART) as CD4 percentage <20% in a child under 18 months old and CD4 percentage <15% in a child over 18 months old [6]. In April 2010 those criteria were expanded to include ART for all HIV-infected infants, children 1–5 years old with CD4 T-cell counts <750 cells/mm^3^ and 5–15 years olds with CD4 T-cell counts <350 cells/mm^3^.

In 2004 the United States President’s Emergency Plan for AIDS Relief (PEPFAR) began assisting the SAG to establish and scale up HIV prevention, care and treatment programs in private, non-government and government-supported clinics through partnerships with various implementing organizations [7]. PEPFAR assists the delivery of antiretroviral treatment (ART) through a range of services depending on the resource needs of the facility. Assistance may include the renovation of facilities, hiring of clinical staff, training and mentoring of clinicians, technical expertise, laboratory services, supply chain strengthening, the provision and distribution of drugs, quality assurance, and monitoring and evaluation activities. Approximately 90% of PEPFAR assistance goes toward government facilities with the remaining 10% reaching private facilities. The South African National Department of Health is responsible for policy development and implementation including determination of the eligibility criteria for treatment initiation. Over the period 2004–2010 the National Department of Health took increasing responsibility for procurement of prophylactic and antiretroviral drugs, reimbursement for the laboratory costs, and supply of the professional workforce.

In 2005, of the approximately 93,000 children in need of ART, less than 12,000 (13%) were receiving treatment [2], [8]. In 2010, 183,000 South African children were estimated to require ART [2]. The SAG set targets to initiate 38,000 new children on ART between April 2010 and March 2011, and have 150,134 children on ART by March 2011 [9].

Using routine program data, we describe increases in pediatric HIV/AIDS treatment and select outcomes in PEPFAR-assisted programs in South Africa from 2004 through 2010.

## Patients and Methods

We reviewed routine quarterly HIV care and treatment program output data submitted by PEPFAR-funded implementing partners, primarily local and international non-governmental organizations who are responsible for the delivery of high-quality HIV care and treatment programs. Those partners submit quarterly data through an open-source web-based platform. Partners reported by healthcare facility the following measures: the number of patients initiating (newly initiated and those transferred in) ART, currently receiving ART, and the cumulative number not receiving ART by age group. Those not receiving ART were defined as those who were seen at a clinic but did not meet eligibility criteria for ART; or had been on ART and categorized as discontinued due to lost-to-follow-up, transferred out, stopped, died or unknown reasons. Data were reviewed for face-validity, trends and logical errors for each submitting partner each quarter by program officials during the reporting period. We could only access data from pediatric HIV programs that were partially or fully supported by PEPFAR. Therefore, we did not include pediatric HIV-infected patients who were cared for by private practitioners or SAG programs not associated with PEPFAR. To assess the reach of the PEPFAR program, we obtained the number of children on ART nationwide from the National Department of Health ART monitoring and evaluation system (a paper-based monthly reporting system) [8].

We completed a retrospective analysis of measures for persons less than 15 years old who ever received ART in PEPFAR-assisted programs in South Africa between October 1, 2004 and September 30, 2010. Detailed data on attrition were only available from April 1, 2008 through September 30, 2009. Using Microsoft Excel**®** and SAS**®** version 9.1 (SAS Institute Inc., Cary, NC), data were analyzed at the end of each of the United States Government fiscal year (October 1-September 30). We calculated monthly averages by dividing the total number for a period by the number of months in that period. We calculated prevalence ratios (PR) and 95% confidence intervals (CI) by age group and continuation on antiretroviral therapy. We estimated the number of years of life added due to treatment of HIV-infected 0–4 year olds, up to 30 years of age. To do that, we used the number of children currently on ART at the end of our study period. Mortality estimates for children on ART and children not on ART were derived from UNAIDS/UNICEF recommendations and other published reviews included in Spectrum Software version 4.351 [10]. We used those estimates to calculate the combined number of years the children would have lived if they were not provided with ART and subtracted it from the number of years those children were expected to live now that they are on ART. To simplify the model the mortality estimates for children not on ART assume only perinatal infection. The mortality estimates account for varying mortality risk in each year of life. We also calculated the number of deaths averted in the first five years of life for the 0–4 year olds currently on PEPFAR-assisted ART at the end of the study period by subtracting the number of deaths estimated for children on ART from those not on ART using mortality estimates from Spectrum [10].

In accordance with United States regulations and international guidelines, the Centers for Disease Control and Prevention and the South African Government determined the review of programmatic data and subsequent analyses to be a non-research activity.

## Results

**Figure 1:**
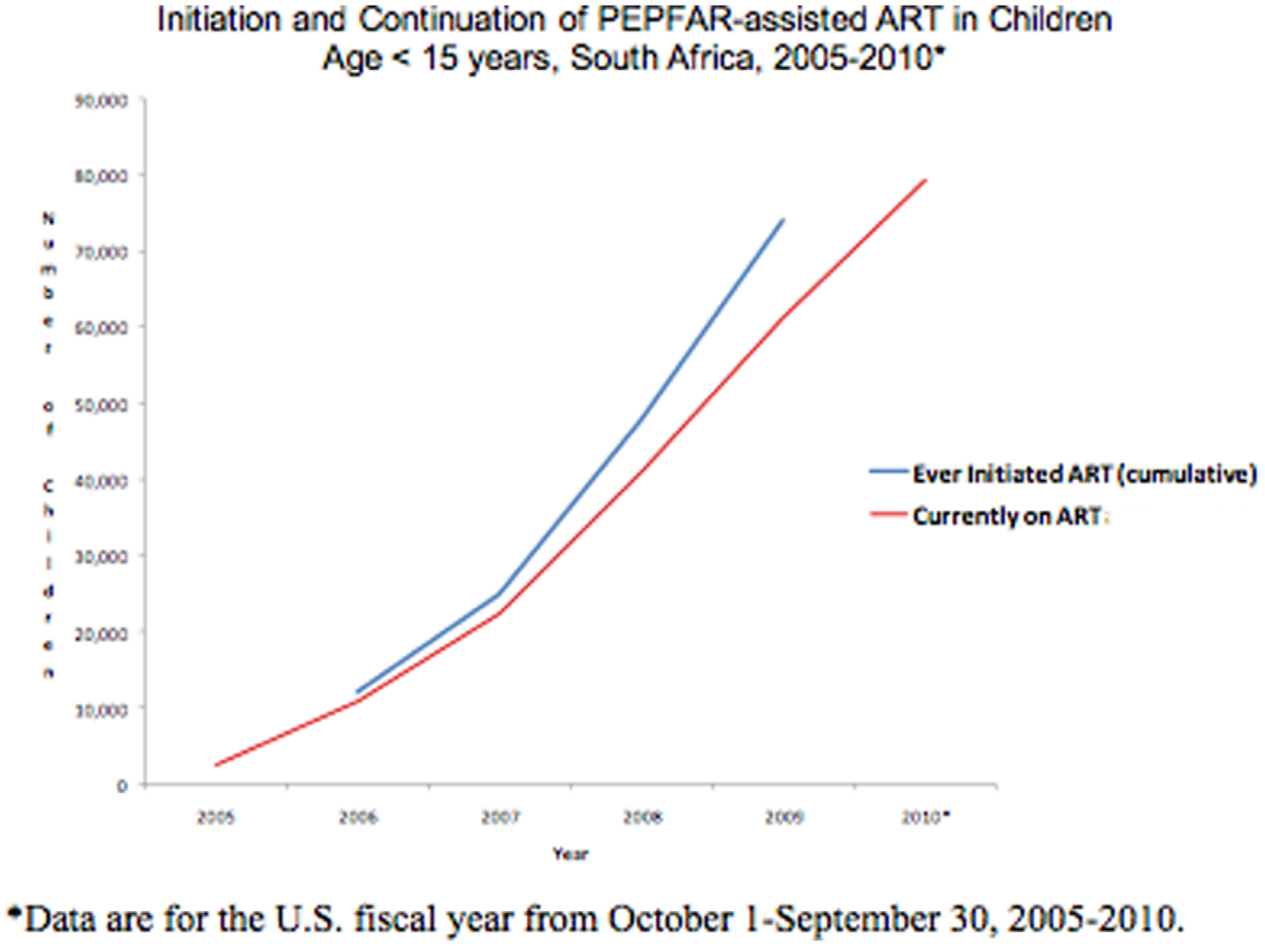
Initiation and Continuation of PEPFAR-assisted Antiretroviral Treatment (ART) in Children, South Africa, 2005–2010.

**Figure 2:**
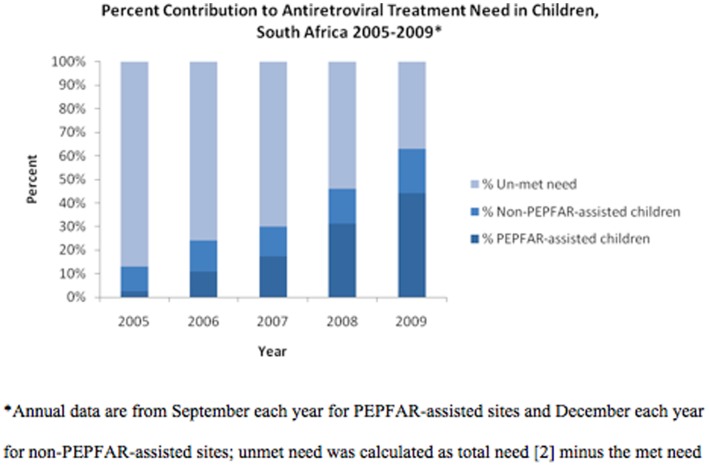
Percent Contribution to Antiretroviral Treatment (ART) Need in Children by PEPFAR Assistance, South Africa 2005–2009.

The number of children ever initiated on PEPFAR-assisted ART increased from 2,412 children in September 2005 to 79,416 children in September 2010, a 32-fold increase (Figure 1). The rapid increase of ART within PEPFAR-assisted programs contributed substantially to expanding coverage from the estimated 12.5% of HIV-infected children in need of ART in 2005 to over 54% in September 2010. As part of the national effort, PEPFAR-assisted programs contributed to 20% of children receiving ART in 2005 and to over 75% of those receiving treatment in September 2009 (Figure 2).

**Figure 3:**
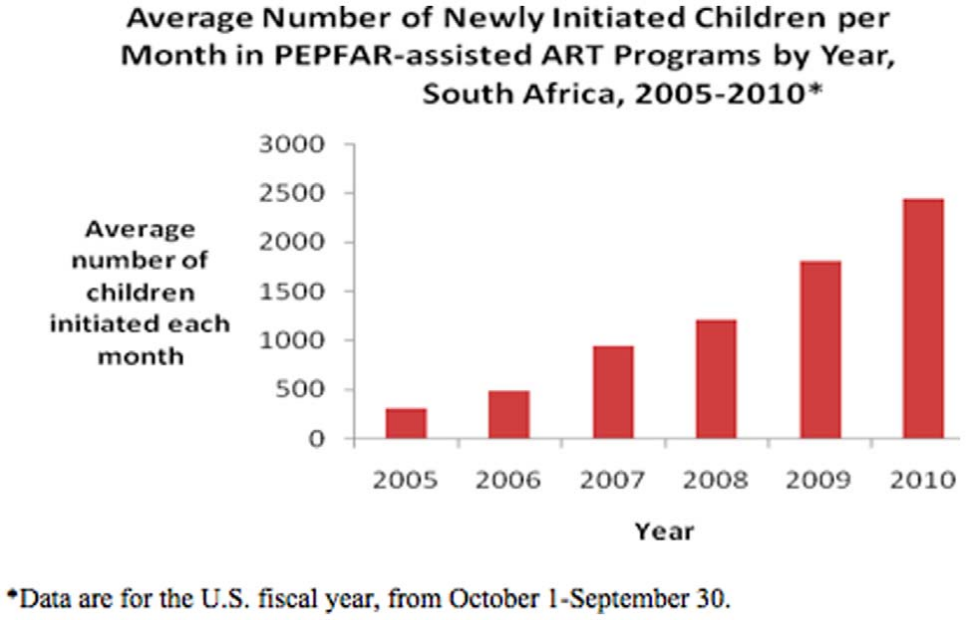
Average Number of Newly Initiated Children per Month in PEPFAR-assisted Antiretroviral Therapy (ART) Programs by year, South Africa, 2005–2010.

The number of children in PEPFAR-assisted programs newly initiating ART per month increased 16-fold from 154 new children per month in 2005 to 2,641 new children per month in 2010 (Figure 3). From March to June 2010, 9,107 children were newly initiated on ART by PEPFAR-assisted programs contributing to 24% of the March 2011 SAG target.

**Figure 4:**
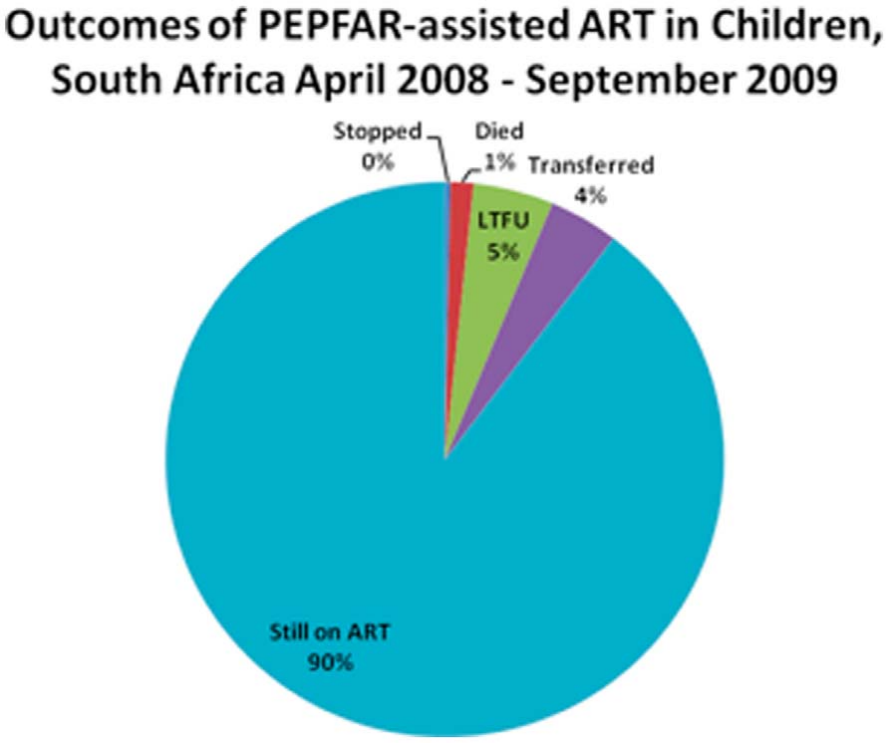
Outcomes of PEPFAR-assisted Antiretroviral Therapy (ART) in Children, South Africa 2008–2009. *Lost to Follow-up (LTFU).

Of the 74,549 children started on ART, 66,870 (89.7%) were retained on ART by the end of September 2009. The attrition of 7,679 children from ART in PEPFAR-assisted program between April 2008 and September 2009 can be attributed to multiple causes: of the 74,549 children who ever initiated ART, 0.3% had stopped treatment (0.28% of 0–4 year olds compared to 0.34% of 5–14 year olds (PR  = 0.83, CI 0.63–1.09)), 4.0% had transferred care (5.0% of 0–4 year olds compared to 3.5% of 5–14 year olds (PR  = 1.41, CI 1.32–1.52)), 1.3% had died (1.6% of 0–4 year olds compared to 1.2% of 5–14 year olds (PR  = 1.32, CI 1.17–1.50)), and 4.6% were lost to follow-up (4.8% of 0–4 year olds compared to 4.6% of 5–14 year olds (PR  = 1.05, CI 0.98–1.12)), (Figure 4). Males were 1.35 times as likely as females to stop treatment (CI 1.04–1.74).

Just over one-third of the 79,416 HIV-infected children on PEPFAR-assisted ART in South Africa is younger than five years old. After removing an additional 8.9% for children who were lost-to-follow-up, transferred, or stopped treatment, a total of 23,875 children remain for analysis. Those children would live for a total of 59,830 years without treatment and 87,378 years with treatment. This suggests that ART has added 27,548 years of life for children under five who were receiving ART in September 2010. Alternatively, in the first five years of life it is estimated that 13,875 of these children would die without treatment as opposed to 8,046 on treatment, suggesting that in the first five years of life, ART averted 5,829 deaths.

## Discussion

The provision of ART to children in South Africa has greatly increased in the past six years through the South African government’s treatment programs and with the assistance of PEPFAR. The 79,416 children currently receiving PEPFAR-assisted ART in September 2010 contribute to over 50% of the March 2011 SAG target of 150,134 children receiving ART. In April 2010, the SAG updated the criteria for ART initiation to: all HIV-infected infants <1 year old, 1–5years olds with CD4 T cell counts <750 cells/mm^3^ and 5–15 year olds with CD4 T cell counts <350 cells/mm^3^ to help prevent high mortality in untreated children [11], [12]. As those guidelines are put into effect, the number of children receiving ART should further increase substantially [13].

Pediatric treatment services are largely centralized (based at district hospitals) at present. In 2006, only 6% of children received ART in community (or primary) health centers in the most populated province, Gauteng [14]. However, it has been shown that provision of ART at the primary healthcare clinic level can result in increased number of patients receiving therapy without compromising quality of care [15]. In April 2010, the SAG prioritized Nurse Initiated and Managed Antiretroviral Treatment (NIMART) to decentralize adult services. The addition of pediatric care and treatment components to the NIMART program could further increase the number of children provided with ART.

Similar to other studies in which an administrative database was used, our study had some limitations. Programmatic measures change frequently, undergo inconsistent quality assurance, and are subject to over- and under-reporting. Due to programmatic changes in data collection procedures, data related to pediatric mortality and treatment drop-out were available for only six of the 24 quarters in the study period, and therefore, might not be generalizable. Additionally, the comparisons of outcomes between children age less than 5 years of age and greater than 5 years of age must be interpreted with caution since those on treatment age greater than 5 years of age have a survival bias. The characteristics of survivors might be different than non-survivors. Regardless, the main findings are likely valid and are consistent with past studies that also showed that loss to follow-up in this population secondary to transfer of care to other treatment providers is an issue, thus necessitating additional efforts to understand why 0–4 year olds are more likely to transfer care or die [21]. Other measures, such as the age of ART initiation, provider type, regimen type and duration, serial CD4 T-cell counts, plasma HIV viral load, hospitalization and mortality, should be routinely collected among the pediatric population in order to discern any differences in clinical outcomes at the national level.

The proportion of children continuing on treatment (89.7%) was better than that of the adult population (77%) in PEPFAR-assisted programs in South Africa for a similar period [16] and on the higher end of other reports on children from sub-Saharan Africa [17]–[19]. Because cumulative attrition data were reported each quarter, there may have been some misclassification of program outcomes due to reporting error. We were limited by the available data in determining factors that might influence retention. Detailed patient data such as, clinical stage of illness, socioeconomic status, migration status and health of primary caregiver along with facility characteristics such as, the size of the facility, ratio of staff-to-patients, urban versus rural setting, transport costs, and average distance from patient’s homes were not available. Further evaluation is needed to understand how those characteristics might contribute to difference in continuation on ART in children.

While the overall reported discontinuation of PEPFAR-assisted treatment was relatively low, the most frequent reason (4.6%) was lost to follow-up, followed by transferred care (4.0%). Current patient monitoring systems for HIV care and treatment in South Africa are unevenly distributed across clinical sites and do not allow for the tracking of individuals at the provincial or national level [20]. A national system utilizing a unique patient identifier could greatly facilitate the care of patients and the monitoring of patient outcomes. However, the system must be designed to ensure patient confidentiality. Furthermore, patient outcomes can be improved by supporting current efforts to initiate an HIV case reporting system.

When estimating number of years of life added for children under five, we only took into account ART administration. Our estimate is conservative in that we did not take into account the impact of pre-ART care interventions, such as nutritional programs and opportunistic infection prophylaxis, which may also lead to reduced mortality [21]–[22]. However, our assumptions did include perinatal transmission for those children not receiving treatment, which is the worst-case scenario and may overestimate mortality. As interventions to prevent mother to child transmission improve, there will be fewer perinatal infections. We also assumed that children on ART began early in life, which may overestimate the number of life-years added by treatment. South African vital statistics demonstrate a trend in declining all-cause mortality in children under five beginning in 2006, after a steady and dramatic increase in mortality from 2001–2006, further supporting the link between ART and reduced mortality [3], [23].

Initiatives to prevent and treat HIV infection are underway in South Africa; however, HIV/AIDS cannot be looked at in isolation without addressing other factors contributing to the increased infant/child mortality, such as infant and child feeding practices and respiratory diseases. The functional separation of services such as Prevention of Mother to Child Transmission programs, immunizations, management of childhood illnesses, and diagnosis of HIV infection leads to decreased continuity of care for children and missed opportunities for intervention resulting in increased morbidity and mortality. Integration of services and increased involvement of community health workers could enhance the probability of achieving national goals [14]. Community health workers help HIV infected patients initiate care at earlier stages and they improve patient retention, especially in rural settings [15]. Also, programs to improve family planning and maternal health are necessary for long-term success with children as the prevalence of HIV infection in child-bearing women is 29% [24] and it is clear that disability and loss of mothers (due to morbidity or mortality) affects the overall welfare of the child.

From 2005–2010, the SAG with assistance from PEPFAR increased ART for children. This collaboration added approximately 27,549 years of life for under-five children currently receiving ART. While that is a major medical achievement, significant challenges lay ahead in terms of reaching the national targets and providing life-long care and treatment. Specific needs to address are: lack of a central system for case reporting, reliable distribution of antiviral and other life-saving drugs to all clinics, decentralization of ART initiation and administration, integrated care, and strengthening the capacity of community-based services. At present, the management of HIV infection necessitates lifelong regular clinical care. Capacitating the South African public medical system and providing care and treatment for HIV-infected children will require increased local community involvement, advocacy, financial investment and political will.
